# Development of a performance measurement system for general practitioners’ office in China’s primary healthcare

**DOI:** 10.1186/s12913-022-08569-z

**Published:** 2022-09-21

**Authors:** Wenjie Ruan, Changming Liu, Xiaoyang Liao, Zhaoxia Guo, Yalin Zhang, Yi Lei, Huadong Chen

**Affiliations:** 1 General Practice Ward/International Medical Center Ward, General Practice Medical Center, West China Hospital, Sichuan University, Chengdu, China; 2 Business School, Sichuan University, Chengdu, 610065 China

**Keywords:** General practice, Performance measurement system, Quality of health service, Focus group method, Delphi method

## Abstract

**Background:**

General practitioners are the main providers of primary care services. To better strengthen the important role of general practitioners in primary healthcare services, China is promoting the general practitioners’ office system. There is a lack of well-accepted methods to measure the performance of general practitioner offices in China. We thus aim to develop a systematic and operable performance measurement system for evaluating the general practitioner’s office.

**Methods:**

We establish an index pool of the performance measurement system of general practitioners’ offices by a cross-sectional study and the literature research method and adopt the focus group method to establish the preliminary system. The Delphi method is then used to conduct three rounds of consultation to modify indices, which aims to form the final indicator system. We determine the weight of each index by the analytic hierarchy process method, which together with the final indicator system constitutes the final performance measurement system. Finally, we select three offices from three different cities in Sichuan Province, China, as case offices to conduct the case study, aiming to assess its credibility.

**Results:**

Our results show that the first office scored 958.5 points, the second scored 768.1 points, and the third scored 947.7 points, which corresponds to the reality of these three offices, meaning that the performance measurement system is effective and manoeuvrable.

**Conclusions:**

Our study provides support for standardizing the functions of China’s general practitioner’s office, improving the health service quality of generalists, and providing a theoretical basis for the standardization of the general practitioner’s office.

**Supplementary Information:**

The online version contains supplementary material available at 10.1186/s12913-022-08569-z.

## Background

The Chinese government attaches great importance to the physical health of the people and has put forward the national strategy of Healthy China 2030, which proposes to provide comprehensive and holistic health services for the population by strengthening the primary health service system and building a team of general practitioners [1]. However, in China, due to the late start of medical development and backward concepts, the uneven distribution of medical resources and the mismatch between supply and demand have led to widespread problems such as an imbalance in supply and demand between doctors and patients and difficulty in seeing a doctor.

General practitioners (also known as family doctors) are the main providers of primary care services [2]. In some developed European countries, general practitioners account for more than 50% of the total number of medical practitioners in the health care system [3]. In China, however, the general practitioner system started late, and the proportion of consultations undertaken by general practitioners is still relatively small [3].

To better strengthen the important role of general practitioners in primary healthcare services, the general practitioners’ office system is being widely implemented across China. The general practitioners’ office is the main venue for family doctors contracted in primary health care institutions to provide compliance services. The contracted family doctors provide comprehensive, continuous and one-stop services, such as basic medical care, public health and health management, to the contracted residents through the general practitioners’ office, including basic public health services, treatment of common diseases and chronic diseases, appointment booking, convenient referral, joint consultation or remote consultation, health assessment, management plan formulation, follow-up visits, and other compliance services [4–6]. The establishment of the general practitioners’ office has a positive effect on promoting the focus of medical and health work as well as resources to be placed more at the grassroots level, achieving basic medical and health services for all, and reducing the problems of expensive and difficult access to health care [7]. However, at present, there are various problems with the construction of general practitioners’ offices in China, such as nonuniform construction standards and service processes, a single medical service model, incomplete medical service content and insufficient service levels of family doctors [8]. Better identification of these problems requires the establishment of a scientific and effective performance measurement system to evaluate the construction of general practitioners’ offices.

The UK government introduced the Quality and Outcomes Framework as a system for assessing performance in 2004 [9]. Grol conducted an international study to determine the priorities of patients in general practice care [10]. Countries such as Austria and Poland have optimized the performance of family doctors, the process of collaborative service and other aspects. Standardized assessments are made in the workplace where family doctors provide health services in terms of basic medical services, quality of care, continuity of care, coordination and safety [11–13]. A study from Switzerland showed that after optimizing the interior complements of the primary care office, the level of medical and health services and patient satisfaction improved [14]. Another study in Switzerland showed that the adjustments and optimization of process elements such as the workflow of primary care office, medical training, and the result elements such as degree of satisfaction to evaluate the performance of office have all been recognized [15]. Additionally, the quality of health services can also be improved by optimizing the physical environment, service mode, team training and communication skills [16].

A systematic and operable performance measurement system for general practitioners’ offices that is suitable for China’s reality can gradually standardize the functions of China’s primary care institutions, improve the health service quality of generalists, and provide a theoretical basis for the standardization of general practitioners’ offices. However, a scientific and effective performance measurement system for evaluating general practitioners’ office has not been established thus far.

The objective of this paper is to develop a reliable and effective performance measurement system for general practitioner’s office. The novelty and contributions of this paper in theory and practice include (1) developing a four-step hybrid approach to construct a performance measurement system, (2) providing a systematic and operable indicator system for evaluating general practitioners’ offices in China, and (3) providing an accessible tool for primary care institutions to improve the quality of healthcare services.

## Methods

A scientific and reliable performance measurement system is developed according to the following four-step hybrid approach. First, we establish an index pool for evaluating the performance of general practitioners’ offices by a cross-sectional study and the literature research method [17]. Second, the focus group method is adopted to organize the personnel who have been engaged in primary care-related work for more than 1 year to carry out three rounds of discussions, based on which a preliminary performance measurement system is established [18]. Third, the Delphi method is then used to conduct three rounds of consultation for general practice experts with more than 10 years of experience in primary care services, based on which index items are modified further to form the final indicator system [19]. Fourth, we determine the weight of each index by the analytic hierarchy process method, which together with the final indicator system constitutes the final performance measurement system [20]. The four steps are detailed as follows.

### Construction of index pool

The literature research method was used to find relevant indicators for evaluating the performance of general practitioners’ offices. We searched for relevant literature before 2022, from several main academic databases (including PubMed, Web of Science, and CNKI). The keywords used for the literature search included primary health care, family practice, general practitioners, community health centers, community health workers, community health services, chronic disease, chronic disease indicators, quality indicators, and health care. We then identified relevant initial indicators from the references found and put them into the index pool.

Next, a cross-sectional study was conducted to collect the configuration and construction requirements of general practitioners’ offices by surveying medical personnel who had been working on primary care services for at least 1 year in primary care institutions of Sichuan Province, China. We designed structured questionnaire based on the current demands of primary care institutions for construction improvement of general practitioners’ offices [8] and sent them to the survey participants by online chatting tools (such as QQ and WeChat groups). The questionnaire is presented in Additional file 1. The questionnaire includes 5 parts:

1. The basic information of the respondent: gender, age, position, educational level, etc.;
2. The configuration requirements of general equipment in a general practitioners’ office;
3. The configuration requirements of the medical equipment in a general practitioner’s office;
4. The requirements of medical personnel in a general practitioner’s office;
5. The primary care services provided in a general practitioner’s office.

Each requirement or service was represented by a certain option. An option was set as a candidate measurement index and put into the index pool if it was chosen by more than 75% of respondents. After removing duplicated indicators, the index pool was finally formed, and the indices in the pool were divided into three groups from structure, process, and outcome dimensions respectively, according to the Structure-Process-Outcome theory [21].

### Construction of the preliminary performance measurement system

Three rounds of expert discussions were conducted using the focus group method, with the participation of medical personnel who were currently working on primary care services for 1 year or more, which aimed to establish the preliminary performance measurement system [22, 23]. The three round of discussions involve 27 individual participants in total. The first two rounds of discussions were conducted online with 15 and 11 participants respectively, and the third was conducted offline with 10 participants. The number of participants chosen are reasonable that (1) it is usually suggested for the focus group method to use no less than 6 members [24], and (2) our experts involved are qualified and have rich experience in primary care services to produce reliable and unbiased consensus to improve a tentative performance measurement system.

The tentative three-level performance measurement system was established based on the index pool obtained in Section 2.1 before the discussions, where each lower-level indicator is subordinate to the upper-level indicator that contains it and the first-level indicators represent three indicator dimensions (i.e., structure, process, and outcome). In each discussion, a brief introduction to this research was given to ensure that the experts understand the discussion goals clearly. Experts were asked to evaluated indices in terms of reasonableness, operability, and importance in each discussion, and their opinions on each index’s appropriateness and suggestions to index changes were collected. In the first-round discussion, the tentative performance measurement system was discussed by experts and was further modified in the last two-round discussions. Each discussion lasted approximately one and a half hours, and the details were recorded and documented. After each discussion, we collated the contents of discussion and then improved the performance measurement system accordingly.

### Modification of index items in the performance measurement system

The preliminary performance measurement system was then modified according to the Delphi method, which was a process combining the benefits of expert analysis with elements of the wisdom of crowds [25]. The consultants consisted of some researchers from Mainland China who had published “General Practitioners’ Office”-related research articles and experts and managers with more than 10 years of primary care service experience from primary healthcare institutions in Sichuan Province, China. The questionnaire was designed based on our research objective, in which options such as importance and operationality were set under each index item in the preliminary performance measurement system. Experts were asked to score each option on each indicator, with the scores quantified by a Likert scale and the full score is 5. The questionnaire also contained an expert authority self-assessment form, which meant that experts were required to score their familiarity with each item and the basis for their judgment. A field of comments at the end of the questionnaire allowed experts to suggest changes to the questionnaire entries.

We conducted the Delphi method repetitively for three rounds, with each round screening the indices of the performance measurement system based on the experts’ scores and comments. In the screening process, indices were selected according to their importance and operability. First, the arithmetic mean value and variation coefficient of importance and operability of each index were calculated. The inclusion criteria included a mean value greater than 4.0 and a coefficient of variation less than 0.25, while the exclusion criteria included a mean value less than 3.0 and a coefficient of variation greater than 0.25. Other indices with mean values between 3.0 and 4.0 needed to be adjusted or deleted after discussion.

In addition, in each round, the authority, motivation and coordination of the experts were evaluated based on their authority self-assessment form, the recovery rate of the questionnaire and whether there were disagreements among the experts. The corresponding indicators for expert evaluation are detailed below:

1. Authority of experts: It is represented by the authority coefficient (*Cr*), which is defined as the average of a judgment basis indicator and a familiarity degree indicator. The values of the two indicators with a value range of [0, 1] can be obtained from the expert authority self-assessment form. A score greater than 0.7 is considered reliable.
2. Motivation of experts: It is represented by the on-time recovery rate and final recovery rate of questionnaire in each round. On-time recovery rate = (number of questionnaires collected on time/number of questionnaires distributed) × 100%; Final recovery rate = (number of questionnaires finally collected/number of questionnaires distributed) × 100%. A score greater than 0.7 indicates a high motivation of experts.
3. Coordination of experts: It represents the consistency degree of experts’ opinions on each index. It is an indicator of the credibility of the results, expressed by the Kendall’s coordination coefficient *W*. The larger the value of W, the higher the expert coordination. The coordination of expert is considered high at *W* > 0.5.

### Determination of index weights for the final performance measurement system

The analytic hierarchy process method was used to measure the weight and combination weight of index items [26]. Three steps of the AHP methodology are presented below:

- Step 1: The first was to define a multilevel hierarchy with items in each level having the same or similar attributes [27]. Based on the previous section, a three-level “hierarchy” was established, which meant that indices with the same magnitude were placed in the same level. For example, first-level indices were placed in the first-level tier.
- Step 2: We then made pairwise comparisons of elements at the given level, which aimed to develop a judgment matrix. After that, the consistency parameter was calculated to check whether the judgment matrix was reasonable.
- Step 3: The eigenvalues of the judgment matrix were calculated to determine the relative priority or weight of each element relative to each element in the hierarchy.
- Step 4: We combined the weights of each index in each level to obtain the final performance measurement system.

## Results

This section presents how the aforementioned method is used to develop the performance measurement system for evaluating general practitioners’ offices in China.

### Index pool for evaluating general practitioners’ office in China

Based on the literature research method, the titles and abstracts of relevant papers were summarized and categorized according to the purpose and theoretical basis of this research. A total of 44 indices were finally selected.

In the cross-sectional research, a total of 1917 questionnaires were collected from medical personnel from 21 cities and prefectures in Sichuan Province, in which 1651 valid questionnaires were identified, and the effective rate was 86.12%. Among the respondents, 1103 (66.8%) were female, and 625 (37.9%) were 36 to 45 years old.

Based on the results of the cross-sectional research, a total of 12 indices were identified, including “appearance of the office”, “office size”, “facilities & equipment”, “interior layout”, “staffing”, “team building”, “content of service”, “construction of information system”, “appointment service”, “referral service”, “management of health profiles”, and “follow-up service”, which are incorporated into the index pool.

After the integration of the results of the literature research and cross-sectional research, 56 index items were initially screened out, and 13 index items with repetitive significance were eliminated. Finally, 42 indices were selected to form the performance measurement system. The index pool is sorted out as follows:

1. Structural dimension (15 indices): “appearance of the office” [14], “construction of information system”, “team building” [28], “sources of funding” [29], “satisfaction” [14], “naming”, “office size”, “facilities & equipment” [14], “interior layout”, “staffing”, “regional healthcare information system” [30], “mode of operation”, “culture building of team” [16], “capacity building of team”, “government input”.
2. Process dimension (16 indices): “basic health care”, “contract service of family doctor” [31, 32], “public provisioning of health services”, “collaborative community-based services” [33], “quality of service”, “telemedicine services” [34], “synergy of government” [35], “general medical services”, “emergency medical services” [36], “health education and advisory services” [37], “health management service”, “appointment service” [38, 39], “pharmaceutical delivery”, “outpatient services”, “medication guidance”, and “service for long-term prescriptions” [37].
3. Outcome dimension (11 indices): “income”, “income of basic health care”, “income of public provisioning of health services”, “effective contract rate”, “compliance rate”, “rate of contract renewal”, “contract rate for key populations”, “rate of hypertension control” [40], “rate of diabetes control” [40], “satisfaction of medical staff”, “client satisfaction” [41].

### Preliminary performance measurement system

To establish a preliminary performance measurement system, three single-focus group discussions were conducted in this stage. The first and second focus groups were conducted online, and the third group was conducted on-site. There were 27 participants, 16 of whom were male (59.26%) and 15 of whom were over 40 years old (55.55%).

The first-round discussion lasted 90 minutes with 15 participants. The experts analyzed and discussed the reasonableness, operability and importance of each index, among which, three first-level indices were determined, including “essential requirement”, “health service”, and “quality assessment”. The second-round discussion consisted of 11 participants and lasted 73 minutes. In this stage, experts focused on the categorization of indices, and discussed the specific meaning of each index. For example, they agreed to use “Capacity building of team” to represent the clinical capacity and communication skills of team members. The third-round discussion lasted 90 minutes with 10 participants. In this discussion, experts revised the indices at all levels. For example, they reached an agreement to remove three redundant indices, including “Self-paying”, “Performance-based priority allocation program” and “Growth rate of the number of receptions”. Finally, three first-level indices, 12 second-level indices, and 31 third-level indices were determined. The resulting preliminary performance measurement system is shown in Additional file 2.

### Modified performance measurement system

**Fig. 1:**
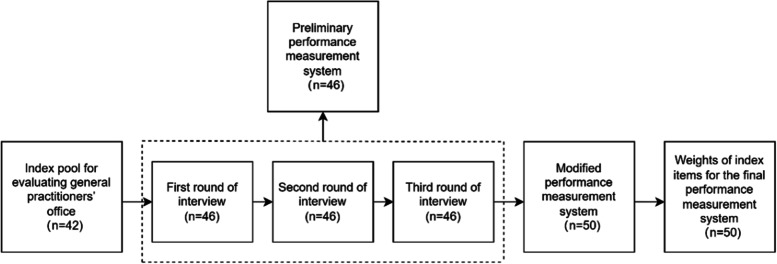
Evolution of the number n of indices obtained in each step

An expert team was formed to modify the preliminary performance measurement system, which consisted of 13 consultants from different medical institutions in Chengdu, Shanghai, Chongqing, Beijing, and Shijiazhuang, with 10 experts (77.0%) over 40 years old, the longest working time of 45 years, an average of 21.46 years, and 7 experts (53.8%) with a master’s degree or above. After three rounds of discussion through the Delphi method, the performance measurement system of the general practitioner’s office was finally constructed, which contained 10 second-level indices and 37 third-level indices. The 10 second-level indices included “appearance of the office”, “construction of information system”, “team building”, “operational mechanisms”, “basic health care”, “contract service of family doctor”, “collaborative community-based services”, “quality of service”, “social assessment”, and “economic efficiency”. The 37 third-level indices are shown in Additional file 3. The modified performance measurement system has a total of 46 indices in levels 1–3. Figure 1 shows the evolution of the number (denoted by n) of indices obtain in each step.

The motivation, authority and coordination of the experts were tested after each round of questionnaire return. After calculation, the motivation coefficients of the experts in the three rounds were all greater than 0.7, indicating a high positive degree, and the results were reliable. The average authority coefficients (*Cr*) of all experts in the first, second and third rounds were 0.9424, 0.9003 and 0.9499, respectively. The authority coefficients (*Cr*) were all greater than 0.7, indicating that the authority of consultants was strong and that the results can be accepted. The coordination coefficient *W* for the indices at level 1–3 were 0.910, 0.890 and 0.864 respectively in round 1 of Delphi method; 0.919, 0.904 and 0.873 respectively in round 2, and 0.918, 0.905 and 0.896 respectively in round 3. All the resulting coordination coefficients were larger than 0.5, showing a high degree of coordination of expert opinions.

### Weights of index items for the final performance measurement system

The results show that the combined weight of basic conditions is 0.2689, the combined weight of health services is 0.3655, and the combined weight of quality performance is 0.3655. The weights of the second- and third-level indices are shown in Additional file 3.

### Case study

We selected three representative general practitioners’ offices with good primary care facilities and services from three cities (i.e., Chengdu, Bazhong, and Deyang) in Sichuan Province, China, as case offices to conduct the field study and to verify feasibility of the proposed performance measurement system. The scorers were managers of each office. The full score of each index is 1. After scoring and scores were assigned with weights, we multiplied each score of indices by 1000 to make the calculation more convenient. We finally added up all scores to calculate the final score.

The results of this research showed that the first office scored 958.5 points, the second scored 768.1 points, and the third scored 947.7 points. According to the scores of the three offices, office 1 and office 3 are relatively high, while office 2 is relatively low. Their differences are mainly reflected in indicators such as regional healthcare information system, mode of operation and incentives. According to the analysis, office 2 is located in northeastern Sichuan, where the economic level is relatively low, and information resources are relatively insufficient. Office 1 is in Shuangliu County of Chengdu. The informatization construction and working model of this county are relatively good, which is basically consistent with the results of the performance using the measurement system developed in this research, indicating that this system can better evaluate the construction of general practitioner’s offices in primary care institutions. In summary, the performance measurement system is effective and manoeuvrable.

## Discussion

The establishment of a performance measurement system is of great importance for the construction of general practitioners’ offices. However, at present, an effective performance measurement system has not been reported for evaluating general practitioner’s offices in China. On the basis of the advanced experience and practices from various countries in evaluating primary care services [12–16], this research is the first to construct a comprehensive and scientific performance measurement system for evaluating the general practitioner’s office from the perspective of general practitioners.

The rationality of the performance measurement system developed is mainly threefold. First, the method for index pool construction is reasonable. Instead of constructing the index pool based only on the existing index database [11], this research constructs the index pool based on both the literature research and the analysis of 1651 quantitative data, which makes the measurement system more manoeuvrable and reliable. Second, our indices are comprehensive and reasonable which cover not only the evaluation of service quality for related diseases but also the evaluation of various basic public health service items stipulated by related authorities in China. Third, the dimensions of our performance measurement system are comprehensive and consistent with the components from the structure-process-outcome theory. It considers not only the process dimension commonly considered in previous related studies, but also structure and result dimensions which have seldom been reported in the literature [42]. The completeness of the constructed performance measurement system is helpful to ensure the rationality of the measurement system.

However, this research has two possible limitations. First, in our literature search and analysis, we have not considered the data from gray literature, newspapers, or reports, which may lead to an incomplete index pool, although our index pool has been validated and confirmed by several rounds of expert consultation. Second, the feasibility of the performance measurement system has only been validated in three cities, which may lead to deviation in results. Our future work will further verify applicability and feasibility of this system in more cities and promote the application of this system in primary medical institutions.

## Conclusion

This research presented a four-step hybrid approach to develop a performance measure system for evaluating general practitioners’ offices. First, we used cross-sectional study and literature research methods to establish an index pool, adopted a focus group method to construct a preliminary performance measurement system and used a Delphi method to modify the preliminary system, and finally constructed a comprehensive, scientific and operable performance measurement system for general practitioners’ offices by using an analytic hierarchy process analysis method to assign weights to indices. The four-step hybrid approach has the strengths of both qualitative and quantitative methods and can be used an effective tool to construct performance measurement system in various areas. The performance measurement system is valuable to assess and establish general practitioners’ offices more effectively, which is important to the primary care service system in China and helpful to improve the delivery of quality primary care services.

## Supplementary Information

**Additional file 1.** Questionnaire for construction of general practitioners’ offices.

**Additional file 2.** Preliminary performance measurement system.

**Additional file 3.** Final performance measurement system.

## Data Availability

The datasets used and analyzed during the current study are available from the corresponding author on reasonable request.
