# Supplementary material for: Development of a performance measurement system for general practitioners’ office in China’s primary healthcare
Source: BMC Health Serv Res. 2022 Sep 21;22:1181. doi: 10.1186/s12913-022-08569-z (PMC9491001; doi:10.1186/s12913-022-08569-z)
Supplement: Supplementary file 1 — Additional file 1. Questionnaire for construction of general practitioners’ offices. [file 12913_2022_8569_MOESM1_ESM.docx]

**Appendix-1 Questionnaire for construction of general practitioners’ offices**

Dear all, in order to further improve the quality of health services of general practitioners’ offices, we invite you to fill out the following questionnaire and give objective suggestions, thank you for your support!

Please select the province, city and region: [fill in the blank] *

_________________________________

Are you from a general hospital, community health center or township health center? [Single choice] *

○ General hospital

○ Community-based health service center

○ Township health center

○ Other

Your gender: [Single choice] *

○ Male

○ Female

Your age [Single choice] *

○ 18 years old-25 years old

○ 26 years old-35 years old

○ 36 years old-45 years old

○ 46 years old - 55 years old

○ 55 years old or older

Your work [Single choice] *

○ Management

○ Clinical

○ Public Health

○ Other

Your education [Single choice] *

○ Junior college

○ Bachelor

○ Master

○ PhD

○ Other

Are you a general practitioner? [Single choice] *

○Yes

○No

First Part: Layout and equipment of the general practitioners’ office

The area of the office [Single choice] *

○ 10-15 m2

○ 15-25 m2

○ 25 m2 or more

The location of the office [Single choice] *

○ Reception area on the first floor

○ Outpatient area

○ Specialized area

The color of the door of the office [Multiple choice] *

○ White

○ Green

○ Blue

The whole color of the office [Single choice] *

○ Milky yellow

○ Light green

○ White

Do you think it is necessary to configure the following equipment to improve the quality of service [Multiple Choice] *

□ Sofa

□ Coffee table

□ Plants

□ Air conditioner

□ Hand washing sink

□ Drinking fountain

□ Landline telephone

□ WIFI

□ Cell phone

□ Other _________________

Do you think it is necessary to configure the following office equipment [multiple choice] *

□ Computer

□ Printer

□ Card reader

□ Electronic sign board

□ IPAD

□ Telephone

□ Desk and chair

□ Other _________________

Second Part: medical equipment

Do you think it is necessary to configure the following medical equipment [Multiple choice] *

□ Electronic sphygmomanometer tied to the upper arm

□ Examination couch

□ Film viewer

□ Wall-mounted or table-type GP diagnostic system

□ Portable pulmonary function instrument

□ Percussion hammer

□ Tuning fork

□ Stethoscope

□ Thermometer

□ Flashlight

□ Tongue depressor

□ Glucometer

□ Measuring device of height and weight

□ Other _________________

Do you think it is necessary to configure the following other equipment [Multiple choice] *

□ Wearable devices (e.g. screening atrial fibrillation)

□ Sensor blood glucose meter

□ Equipment for long range consultation

□ Medical simulation mold

□ Other _________________

Third Part: Staffing

Do you think the staffing should be [single-choice] *

○ Attending physician

○ Attending physician who have been in the profession for more than three years

○ Associate chief physician and above

Do you think it is necessary for the office to hire a full-time physician assistant [Single choice] *

○ Yes

○ No (Please skip to question 21)

Do you agree that physician assistants should do non-consultative work in this office [Single choice] *

○ Yes

○ No

Do you think that physician assistants should meet the following requirements [Single choice] *

○Doctor

○Nurse

○Public attendant

Forth Part: Service content

Is it necessary to use a tablet computer to realize the introduction of service contents [Single choice] *

○ Yes

○ No

Is it necessary to have an appointment function for treatment in the office [Single-choice] *

○ Yes

○ No

Is it necessary to make referrals in the office [Single choice] *

○ Yes

○ No

Is it necessary to manage the health records of contracted patients in the office [Single choice] *

○Yes

○No

Is it necessary to carry out subsequent visit in the office [Single choice] *

○Yes

○No

Section 5: Other suggestions

Other suggestions [fill-in-the-blank]

_________________________________
