# Supplementary material for: Development of a performance measurement system for general practitioners’ office in China’s primary healthcare
Source: BMC Health Serv Res. 2022 Sep 21;22:1181. doi: 10.1186/s12913-022-08569-z (PMC9491001; doi:10.1186/s12913-022-08569-z)
Supplement: Supplementary file 2 — Additional file 2. Preliminary performance measurement system. [file 12913_2022_8569_MOESM2_ESM.docx]

**Appendix-2 Preliminary performance measurement system**

| First-Level Index | Second-Level Index | Third-Level Index |
| --- | --- | --- |
| Essential requirement | Appearance of the office | Naming |
|  |  | Facilities and equipment |
|  |  | Office size |
|  |  | Interior layout |
|  | Construction of information system | Regional healthcare information system |
|  |  | Intelligent device |
|  | Team building | Staffing |
|  |  | Mode of operation |
|  |  | Capacity building of team |
|  |  | Culture building of team |
|  | Sources of funding | Government input |
|  |  | [Investment in social capital](https://onlinelibrary.wiley.com/doi/abs/10.1111/j.1728-4457.1999.00001.x) |
| Health service | Basic health care | General medical services |
|  | Emergency medical services | Emergency medical services |
|  | Contract service of family doctor | Health education and advisory services |
|  |  | Health management service |
|  |  | Priority appointment service |
|  |  | Two-way referral service |
|  |  | Pharmaceutical delivery |
|  | Public provisioning of health services | Public provisioning of health services |
|  | [Collaborative community-based](https://linkspringer.fenshishang.com/article/10.1186/1752-4458-3-27) services | Collaboration with resident council |
| Quality assessment | Quality of service | Effective contract rate |
|  |  | Compliance rate |
|  |  | Rate contract renewal |
|  |  | Contract rate for key populations |
|  |  | Rate of hypertension control |
|  |  | Rate of diabetes control |
|  | Satisfaction | Satisfaction of medical staff |
|  |  | Client satisfaction |
|  | Income | Income from basic health care |
|  |  | Income from contract service |
