# Supplementary material for: Development of a performance measurement system for general practitioners’ office in China’s primary healthcare
Source: BMC Health Serv Res. 2022 Sep 21;22:1181. doi: 10.1186/s12913-022-08569-z (PMC9491001; doi:10.1186/s12913-022-08569-z)
Supplement: Supplementary file 3 — Additional file 3. Final performance measurement system. [file 12913_2022_8569_MOESM3_ESM.docx]

**Appendix-3** **Final performance measurement system**

| First-Level Index | Weights | Second-Level Index | Weights | Third-Level Index | Weights |
| --- | --- | --- | --- | --- | --- |
| Essential requirement | 0.2689 | Appearance of the office | 0.0481 | Naming of GPO | 0.0097 |
|  |  |  |  | Facilities and equipment in GPO | 0.0187 |
|  |  |  |  | Size of GPO | 0.0099 |
|  |  |  |  | Interior layout of GPO | 0.0099 |
|  |  | Construction of information system | 0.0734 | Regional healthcare information system | 0.0482 |
|  |  |  |  | Intelligent device | 0.0253 |
|  |  | Team building | 0.0799 | Staffing | 0.0304 |
|  |  |  |  | Service capacity building of team | 0.0304 |
|  |  |  |  | Team cohesion | 0.0191 |
|  |  | Operational mechanisms | 0.0675 | Mode of operation | 0.0246 |
|  |  |  |  | sources of funding | 0.0161 |
|  |  |  |  | Incentives | 0.0268 |
| Health service | 0.3655 | Basic health care | 0.1448 | General medical services | 0.0555 |
|  |  |  |  | Emergency medical services | 0.0378 |
|  |  |  |  | Outpatient services | 0.0270 |
|  |  |  |  | Telemedicine services | 0.0245 |
|  |  | Contract service of family doctor | 0.1448 | Advisory services | 0.0234 |
|  |  |  |  | Health management service | 0.0233 |
|  |  |  |  | Priority appointment service | 0.0189 |
|  |  |  |  | Two-way referral service | 0.0171 |
|  |  |  |  | Pharmaceutical delivery | 0.0142 |
|  |  |  |  | Accessibility of health services | 0.0160 |
|  |  |  |  | Personalized service | 0.0107 |
|  |  |  |  | Public provisioning of health services | 0.0213 |
|  |  | [Collaborative community-based](https://linkspringer.fenshishang.com/article/10.1186/1752-4458-3-27) services | 0.0760 | Collaboration with resident council | 0.0386 |
|  |  |  |  | Service of social worker | 0.0374 |
| Effectiveness assessment | 0.3655 | Quality of service | 0.1323 | Effective contract rate | 0.0236 |
|  |  |  |  | Compliance rate | 0.0217 |
|  |  |  |  | Rate of contract renewal | 0.0199 |
|  |  |  |  | Contract rate for key populations | 0.0217 |
|  |  |  |  | Rate of hypertension control | 0.0217 |
|  |  |  |  | Rate of diabetes control | 0.0236 |
|  |  | Social assessment | 0.1216 | Satisfaction of medical staff | 0.0416 |
|  |  |  |  | Client satisfaction | 0.0416 |
|  |  |  |  | Awareness of services provided by GPO | 0.0383 |
|  |  | Economic efficiency | 0.1117 | Volume of basic health care | 0.0535 |
|  |  |  |  | Volume of contracted services | 0.0582 |
